# Correlations of Corneal Spherical Aberration with Astigmatism and Axial Length in Cataract Patients

**DOI:** 10.1155/2019/4101256

**Published:** 2019-09-10

**Authors:** Min Zhang, Dongjin Qian, Qinghe Jing, Jiahui Chen, Michael Deng, Yongxiang Jiang

**Affiliations:** ^1^Department of Ophthalmology and Vision Science, Eye and ENT Hospital of Fudan University, Shanghai, China; ^2^NHC Key Laboratory of Myopia (Fudan University), Laboratory of Myopia, Chinese Academy of Medical Sciences, Shanghai, China; ^3^Key Laboratory of Visual Impairment and Restoration of Shanghai, Shanghai, China

## Abstract

**Purpose:**

To clarify the distribution of corneal spherical aberrations (SAs) in cataract patients with different corneal astigmatism and axial length.

**Setting:**

Department of Ophthalmology and Vision Science of the Eye and ENT Hospital of Fudan University, Shanghai, China.

**Design:**

Retrospective case series.

**Methods:**

The axial length, corneal SAs, and other corneal biometrics were collected in cataract patients with Pentacam HR and IOLMaster 500. The statistical analysis of the corneal SAs was based on the stratification of axial length and anterior corneal astigmatism.

**Results:**

In total, 6747 eyes of 6747 patients were recruited, with 2416 eyes (58.17 ± 16.81 years old) in the astigmatism group (anterior corneal astigmatism ≥1 D) and others (61.82 ± 12.64 years old) in the control group. In patients with astigmatism <2 D, the total and anterior SAs decreased as the axial length increased (*P* < 0.001). The total corneal SAs of patients with astigmatism of 2-3 D stabilized at around 0.29 *μ*m, whereas those of patients with anterior corneal astigmatism ≥3 D tended to be variable. Age and anterior corneal astigmatism had positive and negative effects, respectively, on SA in the regression model.

**Conclusions:**

Axial length has a negative effect on the anterior and total corneal SAs, which stabled around 0.33 *μ*m and 0.30 *μ*m in patients with axial length of ≥26 mm, respectively. Individualized SA adjustments are essential for patients undergoing aspheric toric IOL implantation with preoperative anterior corneal astigmatism of 1-2 D or ≥3 D. Toric IOLs with a negative SA of −0.20 *μ*m are recommended for patients with anterior corneal astigmatism of 2-3 D if no customized therapy is warranted.

## 1. Introduction

Intraocular lenses (IOLs) have been designed for and are implanted into aphakic eyes to substitute for natural lenses, partly to rectify the spherical refractive power in the ocular optical system. Further advances have been made to restore the ocular physiological status: multifocal IOLs for both distance and near vision, toric IOLs to compensate for corneal primary astigmatism, and aspheric IOLs for the correction of corneal spherical aberrations (SAs).

Astigmatism of order 2 is a low-order aberration and SA is a high-order aberration. Nowadays other astigmatism belonging to high-order aberrations were beyond the correction of both IOLs and glasses. So only astigmatism of order 2 was studied and is shortened as astigmatism in this article. Postoperative residual ocular astigmatism and SAs lead to halo or other visual complaints and worsen the optical performance, even when the best-corrected visual acuity is good. The overall prevalence of astigmatism ranges from 86.8% to 99% [1]. A considerable proportion of these eyes in patients with cataract require correction of astigmatism (43.9% of patients with corneal astigmatism of ≥1.0 D in Southern China, 46.70% in Northern China, 37.80% in Thailand, and 40.41% in the United Kingdom), which can be effectively accommodated by the cylindrical power of the toric IOLs available, as demonstrated in previous clinical studies [1–10]. The correction of SA is also widely performed and is clinically important [11, 12].

When an aspheric toric IOL is required, the correction of astigmatism is usually considered first [12, 13]. However, because different aspheric modifications can be made with the aspheric toric IOLs currently available on the market from different companies, which have different aspheric values, postoperative ocular aberrations can be rectified and the correct selection of the optical parameters of the IOL is important.

Therefore, in this study, we (1) clarify the differences in the corneal biometrics of patients when the cutoff point for defining preoperative corneal astigmatism is 1 D, (2) present the total and anterior corneal SA states associated with different astigmatism levels and axial length levels, and (3) identify the main factors associated with SAs, especially the statistical correlations between astigmatism and SA. Some recommendations are also made for the proper correction of SAs using toric IOLs with an aspheric design.

## 2. Methods

In this retrospective case series, we recruited patients scheduled for cataract surgery between September 29, 2016, and August 15, 2018, at the Eye and ENT Hospital of Fudan University, Shanghai, China. The main inclusion criteria and exclusion criteria were described in our previous study [14]. This study was approved by the Human Research Ethics Committee of the Eye and ENT Hospital of Fudan University, and adhered to the tenets of the Declaration of Helsinki. Written informed consent was provided by all the patients.

All ocular data were collected, defined, and presented following the methods of Zhang et al. [14], using the rotating Scheimpflug camera (Pentacam HR; Oculus, Wetzlar, Germany) and the partial coherence interferometry (IOLMaster 500; Carl Zeiss Meditec, Jena, Germany). These data included the anterior corneal astigmatism, central corneal thickness (CCT), anterior corneal eccentricity and the SAs of the total cornea (total SA), anterior corneal surface (anterior SA), posterior corneal surface (posterior SA), index of surface variance (ISV), index of vertical asymmetry (IVA), keratoconus index (KI), center keratoconus index (CKI), index of height asymmetry (IHA), and index of height decentration (IHD). All 6 conic coefficients (ISV, IVA, KI, CKI, IHA, and IHD) were collected with a cornea scan of 8 mm diameter, whereas others were collected with a cornea scan of 6 mm diameter centered at the corneal apex under the automode of Pentacam. Corneal astigmatism was divided into with the rule (WTR), against the rule (ATR), and oblique astigmatism according to the steep corneal meridian [14].

All patients were divided into four levels based on their anterior corneal astigmatism, with cutoffs = 1 D, 2 D, and 3 D. To identify practical and operational parameters for clinical IOL selection, all the patients were also stratified into seven levels according to their axial length: <20 mm, 20–22 mm (20 mm included while 22 mm not, similarly hereinafter), 22–24.5 mm, 24.5–26 mm, 26–28 mm, 28–30 mm, and ≥30 mm. This resulted in a total of 28 individual groups, with two preliminary stratifications (by anterior corneal astigmatism and axial length). In those patients with astigmatism ≥1 D (including the 1-2 D, 2-3 D, and ≥3 D levels), summarized as the “astigmatism group,” astigmatism correction was considered when planning their surgery. Patients with astigmatism <1 D were considered as the control group.

## 3. Statistical Analysis

To avoid any possible contralateral effect, only one eye of each cataract patient was enrolled in this retrospective clinical study.

All continuous data are shown as mean ± standard deviation (SD). The Kolmogorov–Smirnov test was used to assess the normality of the distributions of continuous data. Because the sample of patients with axial length of <20 mm was small (*n* = 7), this group was removed from subsequent analyses, and only 24 cross-groups were analyzed. One-way analysis of variance (ANOVA) was used to compare the continuous variables among the 24 cross-groups and two-way ANOVA was used to compare the four astigmatism levels and six axial length groups twice, once when the data were stratified by axial length and then again when the data were stratified by astigmatism levels. When either the astigmatism level or axial length was taken as the stratification factor, the other was considered to be the explanatory variable. If a significant difference was detected, a further post hoc multiple comparison test with Bonferroni correction was performed to identify the exact level making the difference. Pearson's *χ*^2^ test was used to compare categorical items among the WTR, ATR, and oblique astigmatism groups. Pearson *r* correlation analyses were used to explore the relationship between corneal biometrics with astigmatism and axial length. A multiple regression analysis was used to explore the exact statistical contributions of the explanatory variables to the corneal SAs adjusted by age as described before [14], in the astigmatism group, the control group, and all the enrolled patients. All data were analyzed with SPSS 23.0 (SPSS, IBM Corp., Armonk, NY, USA). *P* < 0.05 was considered to indicate statistical significance.

## 4. Results

In total, 6747 eyes of 6747 patients were recruited in this study, among which 2416 eyes had anterior corneal astigmatism of ≥1 D. The numbers of patients in the cross-groups (axial length × astigmatism) are presented in Table 1.

The demographic data, corneal biometric data, and axial length of these patients are shown in Table 2 and Supplementary Material Table 1, with comparisons between the two groups. The average age of the patients in the astigmatism group was lower than that in the control group (58.17 ± 16.81 vs. 61.82 ± 12.64 years, respectively, *P* < 0.001). In addition, several statistically significant correlations of the corneal biometrics were found with the axial length and the anterior corneal astigmatism (Supplementary Material Table 2). The compositions of the astigmatism types varied with the different astigmatism levels and axial length (both *P* < 0.001; Supplementary Material Tables 3 and 4).

**Figure 1:**
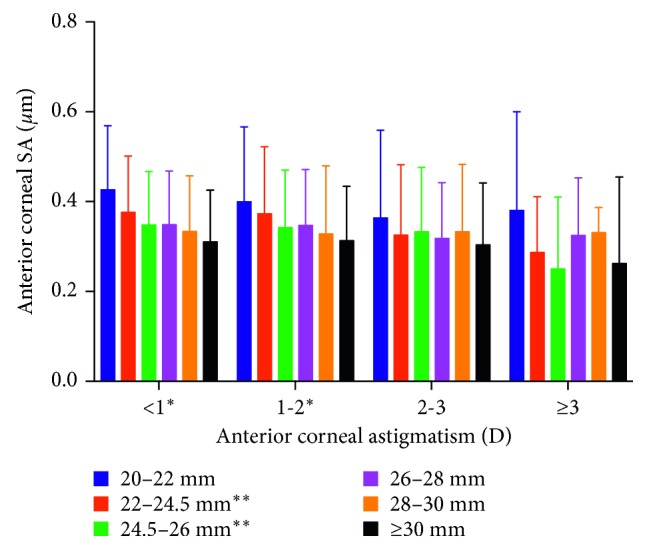
Anterior corneal SA values among cross-groups. Anterior SA = anterior corneal spherical aberration. ^*∗*^*P* < 0.001 among the different axial length levels when the astigmatism levels were taken as the stratification factor. ^*∗∗*^*P* < 0.001 among the different astigmatism levels when the axial length groups were taken as the stratification factor.

**Figure 2:**
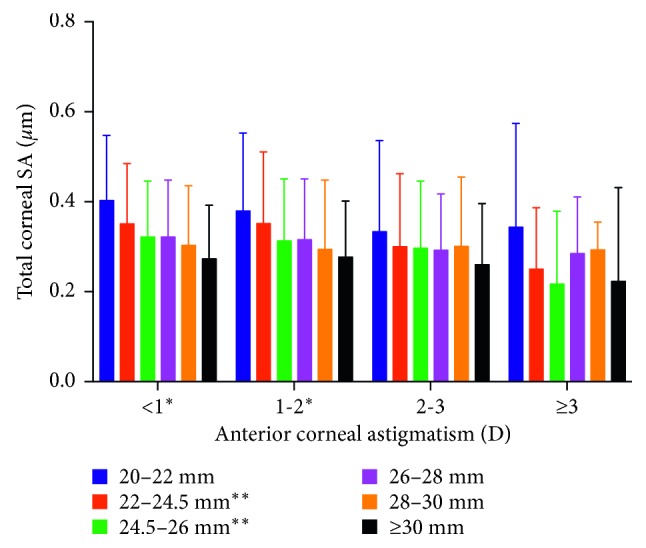
Total corneal SA values among cross-groups. Total SA = total corneal spherical aberration. ^*∗*^*P* < 0.001 among the different axial length levels when the astigmatism levels were taken as the stratification factor. ^*∗∗*^*P* < 0.001 among the different astigmatism levels when axial length levels were taken as the stratification factor.

The distributions of anterior corneal SA and total corneal SA are presented in Figures 1 and 2, respectively. Gradual step-down trends in the anterior and total corneal SAs were detected as the axial length increased in patients with astigmatism <1 and 1-2 D. Although no statistically significant differences in SA were detected as axial length increased in patients with astigmatism >3 D, as mentioned above, the figures show a very variable pattern in these patients. The mean values for anterior, posterior, and total SAs differed significantly among the 24 cross-groups (all *P* < 0.001). Both the anterior and total SAs differed significantly among different axial lengths in patients with astigmatism <1 D or 1-2 D (all *P* < 0.001). Both the total SAs and anterior SAs showed significantly different astigmatism levels in patients with axial length of 22–24.5 mm and 24.5–26 mm. Further analysis showed that the anterior SA differed significantly between patients with axial length of 20–22 mm and those with axial length of 22–24.5 mm (*P* < 0.001) and between those with axial length of 22–24.5 mm and those with axial length of 24.5–26 mm (*P* = 0.002) in the astigmatism <1 D group. The differences in total SA between the following pairs were also statistically significant: axial length of 20–22 mm and 22–24.5 mm in astigmatism <1 D level (*P* < 0.001); axial length of 22–24.5 mm and 24.5–26 mm in astigmatism <1 D level (*P* = 0.002); axial length of 22–24.5 mm and 24.5–26 mm in astigmatism 1–2 D level (*P* = 0.035); and astigmatism of 1-2 D and 2-3 D in the axial length 22–24.5 level (*P* = 0.010). All these data indicate that, in patients with axial lengths ≥26 mm and astigmatism ≥2 D, the total SA and anterior SA varied only slightly. The mean values for total anterior corneal SA could then be calculated, and were 0.29 *μ*m and 0.32 *μ*m in patients with astigmatism of ≥2 D, and 0.30 *μ*m and 0.33 *μ*m in patients with axial length of ≥26 mm, respectively.

In multiple linear regression analyses of the astigmatism group, the control group, and the whole study population, age correlated positively with total SA and posterior SA (all *P* < 0.001; Table 3). When adjusted for age, the axial length correlated negatively with the total SA and posterior SA in the regression models (all *P* < 0.001; Table 3). Based on the absolute value of beta, which represents the corresponding variable's relative contribution in the regression model, age was weighted 3-4 times more strongly than axial length.

A shift from ATR to WTR with age was also detected (Supplementary Material Table 5), which is consistent with previous findings [15, 16].

## 5. Discussion

Phacoemulsification combined with IOL implantation is now much more than a sight-restoring surgery and is indeed a refractive surgery, with the aims of excellent spectacle-independent optical performance and less expensive visual improvement [1, 17].

Postoperative aberrations negatively affect ocular outcomes, which range from halo in both high-contrast and ideal lighting conditions to impaired low-contrast or high-contrast visual acuity when the magnitude of the aberration is large. Among these outcomes, astigmatism and primary SA are the most important low-order and high-order aberrations, respectively, and both are highly prevalent worldwide [18, 19].

The amplitudes of all corneal aberrations, presented as Zernike polynomial coefficients, are affected by the size, shape, and compositional distribution of the cornea [20]. Therefore, corneal astigmatism and corneal SAs must sometimes be revised at the same time. In refractive cataract surgery, toric IOLs are required if patients have an expected postoperative corneal astigmatism of >0.75 D or preoperative corneal astigmatism of >1.00 D. Toric IOLs with SAs of 0, −0.1, −0.18, −0.20, and −0.27 *μ*m are available in clinical practice. When a preoperative corneal SA is identified and individualized correction is possible, the appropriate toric IOL must be selected from among those with different aspheric values.

The careful design of IOLs that considers both a patient's astigmatism and SA is important in achieving the best surgical effect. However, previous studies have only compared the visual outcomes of spherical toric IOL implantation and aspheric toric IOL implantation [12, 13] or aspheric toric IOL implantation and aspheric nontoric IOL implantation [21]. To our knowledge, no research has focused on the role of preoperative examinations in design strategies, so we undertook such an analysis.

Both the anterior corneal surface and the internal optics (the posterior corneal surface and the crystalline lens) contribute to the wavefront aberrations passing through the eye [22]. Crystalline lenses must be removed in cataract surgery and only the corneal properties need be documented preoperatively. Because the effect of posterior corneal astigmatism is much smaller than that of anterior corneal astigmatism, we did not analyze it here. Extreme myopic astigmatism and oblique astigmatism, such as keratoconus [23], are also beyond the scope of this study.

The implantation of toric IOLs is reported not only to compensate for ocular astigmatism but also to reduce any SA [24, 25]. Uncorrected astigmatism reduces the small visual benefit possible by correcting ocular SA with soft contact lenses [26]. However, a postoperative net SA of +0.1 *μ*m is recommended because it allows better contrast sensitivity and an extended depth of focus compared with the aberration-free condition [27–31].

Of the 2416 eyes with astigmatism >1 D (astigmatism group) analyzed in this study, 1983 (82.08%) had astigmatism of 1–2 D, which was the level of most patients in the astigmatism group. At this astigmatism level, the anterior and total SAs were found to vary among the different axial length groups (*P* < 0.001). When the patients were stratified according to axial length, in those with axial length of 22–24.5 and 24.5–26 mm, SA differed according to the level of astigmatism (both *P* < 0.001). The patients in these two axial length groups comprised a large proportion of the cataract population (4629/6747 = 68.60%). This suggests that it is clinically essential to assess the preoperative SA and determine the SA required for correction because these patients constitute such a large proportion of the clinic population.

Although astigmatism of 1-2 D requires individualized SA correction when the astigmatism was ≥2 D, SA tended to be stable (no significant difference in SA was detected in patients with astigmatism of 2–3 D or >3 D; also see Figures 1 and 2). The mean value of the total corneal SA of patients with anterior corneal astigmatism ≥2 D was 0.29 *μ*m. To achieve a postoperative ocular SA of +0.10 *μ*m, toric IOLs with a negative SA of −0.20 *μ*m should be selected. Because of the small group size of ≥3 D astigmatism subgroup (82 eyes) compared with 2–3 D astigmatism subgroup (351 eyes), the 0.32 *μ*m mean total corneal SA in patients with astigmatism of ≥3 D and the not small standard deviation values of total corneal SA in this group (see the ≥3 D astigmatism subgroup in Figure 2), we suggest the selection of −0.20 *μ*m toric IOLs only for patients with astigmatism of 2-3 D and individualized design for those with astigmatism of ≥3 D.

We used multiple regression analyses to identify the factors associated with corneal SAs. It was no surprise to find that astigmatism did not contribute to either the anterior or total corneal SA in the regression models because Miller et al. reported that they observed no association between elevated astigmatism and SA [32]. The negative coefficients for axial length in the regression were consistent with the decreasing trend in SA values as the axial length increased in patients with astigmatism of 1-2 D (axial length < 20 mm was omitted from the analysis because the population was small).

Age was a factor positively affecting SAs, consistent with previous findings [33, 34]. Other corneal biometrics are also reported to correlate with age [35]. Because the members of the astigmatism group were significantly younger than those in the control group, the differences in the corneal biometrics of the two groups can be partly attributed to age, but age does not fully account for the negative role of axial length in the regression model.

There were some limitations in our study. First, posterior corneal astigmatism was not taken into account because of its small magnitude [36], and total corneal astigmatism was replaced with anterior corneal astigmatism in the statistical analyses. The anterior-posterior astigmatism axis, the magnitude of posterior astigmatism, and keratometric astigmatism all lead to estimation errors [37], and this caused the corneal astigmatism values to be overestimated in this study [38, 39]. Second, only IOLMaster was used to measure axial length. IOLMaster does not have a nonaccommodative fixation target and tends to provide inaccurate (usually shorter) axial length in subjects with small pupils. Third, we did not consider dry eye conditions in this analysis (although we excluded patients with dry eye disease). However, the presence of dry eye conditions in individual patients would have affected the accuracy of our topographic results, and therefore the analytical results [40]. Fourth, no vector analysis was performed because no decomposed corneal astigmatism J0 and J45 values were available because of the limitations of our devices [19]. Finally, no white-to-white distance was measured in patients with large and small corneas, which reportedly influence surgically induced astigmatism and postoperative astigmatism [41]. Because of the retrospective design and no postoperative data available, more comprehensive study designs and data collection methods are required in future prospective studies.

## 6. Conclusions

The axial length had a negative effect on the anterior and total corneal SAs, which stabled around 0.33 *μ*m and 0.30 *μ*m in patients with axial length of ≥26 mm, respectively. The anterior corneal SA of patients with anterior corneal astigmatism <2 D decreased as the axial length increased. The total corneal SA of patients with anterior corneal astigmatism of 2-3 D stabilized at around 0.29 *μ*m, whereas those with anterior corneal astigmatism of ≥3 D tended to be variable. Care should be taken when designing the correction of ocular SAs in patients undergoing aspheric toric IOL implantation. Individualized SA adjustments are essential for patients with anterior corneal astigmatism of 1-2 D or ≥3 D. Toric IOLs with a negative SA of −0.20 *μ*m are recommended for patients with anterior corneal astigmatism of 2-3 D if customized therapy is not warranted.

## Figures and Tables

**Table 1:** Numbers of patients in seven axial length categories and four astigmatism groups.

Count (percentage in all)		Axial length (mm)							Total
		<20	20–22	22–24.5	24.5–26	26–28	28–30	≥30	
Astigmatism (D)	<1	5 (0.07%)	209 (3.10%)	2690 (39.87)	530 (7.86%)	380 (5.63%)	221 (3.28%)	296 (4.39%)	4331 (64.19%)
	1-2	1 (0.01%)	76 (1.13%)	907 (13.44%)	289 (4.28%)	264 (3.91%)	198 (2.93%)	248 (3.68%)	1983 (29.39%)
	2-3	1 (0.01%)	22 (0.33%)	121 (1.79%)	51 (0.76%)	55 (0.82%)	39 (0.58%)	62 (0.92%)	351 (5.20%)
	≥3	0 (0%)	6 (0.09%)	25 (0.37%)	16 (0.24%)	14 (0.21%)	7 (0.10%)	14 (0.21%)	82 (1.22%)

Total		7 (0.10%)	313 (4.64%)	3743 (55.48%)	886 (13.13%)	713 (10.57%)	465 (6.89%)	620 (9.19%)	6747

**Table 2:** Demographic data of the astigmatism group and the control group.

Mean ± SD	Astigmatism group	Control group	*P* value
Eyes	2416	4331	—
Age (years)	58.17 ± 16.81	61.82 ± 12.64	<0.001^*∗*^
Gender (male/Female)	1024/1392	1834/2497	0.976^*∗∗*^
CCT (mm)	538.85 ± 31.56	538.29 ± 31.66	0.486^*∗*^
Axial length (mm)	25.72 ± 3.18	24.63 ± 2.66	<0.001^*∗*^

SD = standard deviation; CCT = central corneal thickness. ^*∗*^Independent two-sample *t*-test; ^*∗∗*^Pearson's *χ*^2^ test.

**Table 3:** Results of multiple linear regressions^*∗*^ of the total corneal spherical aberrations and anterior corneal spherical aberrations in the astigmatism group, the control group, and the total enrolled patients.

		Astigmatism group			Control group			Total		
		Coefficient	Beta	*P* value	Coefficient	Beta	*P* value	Coefficient	Beta	*P* value
Total SA	Age	0.005	0.521	<0.001	0.005	0.444	<0.001	0.005	0.478	<0.001
	AL	−0.007	−0.139	<0.001	−0.008	−0.155	<0.001	−0.007	−0.147	<0.001
	CCT	—	—	—	<0.001	−0.027	0.045	0	−0.027	0.009

Anterior SA	Age	0.004	0.445	<0.001	0.004	0.361	<0.001	0.004	0.4	<0.001
	AL	−0.005	−0.117	<0.001	−0.007	−0.148	<0.001	−0.006	−0.135	<0.001
	CCT	<0.001	−0.056	0.002	<0.001	−0.051	<0.001	<0.001	−0.053	<0.001

^*∗*^Anterior corneal astigmatism was removed as a factor in all regressions. Beta = standardized coefficient; total SA = total corneal spherical aberration; anterior SA = anterior corneal spherical aberration; AL = axial length; CCT = central corneal thickness.

## Data Availability

More information about ocular statistical results is accessible in the supplementary materials. The raw data used to support the findings of this study have not been made available because of privacy policies.

## Supplementary Materials

Supplementary Materials Supplementary Material Table 1 presented the conic coefficients of cataract patients with comparisons between the two groups. Supplementary Material Table 2 presented the correlations of the corneal biometrics with the axial length and the anterior corneal astigmatism. Supplementary Material Tables 3 and 4 indicated the compositions of the astigmatism types with the different astigmatism levels and axial length. Supplementary Material Table 5 showed the differences in the proportions of WTR, ATR, and oblique astigmatism in different age levels, indicating a shift from ATR to WTR with age.
