# Supplementary material for: Correlations of Corneal Spherical Aberration with Astigmatism and Axial Length in Cataract Patients
Source: J Ophthalmol. 2019 Sep 10;2019:4101256. doi: 10.1155/2019/4101256 (PMC6754902; doi:10.1155/2019/4101256)
Supplement: Supplementary Materials — Supplementary Material Table 1 presented the conic coefficients of cataract patients with comparisons between the two groups. Supplementary Material Table 2 presented the correlations of the corneal biometrics with the axial length and the anterior corneal astigmatism. Supplementary Material Tables 3 and 4 indicated the compositions of the astigmatism types with the different astigmatism levels and axial length. Supplementary Material Table 5 showed the differences in the proportions of WTR, ATR, and oblique astigmatism in different age levels, indicating a shift from ATR to WTR with age. [file 4101256.f1.docx]

Supplementary Material Table 1. Comparison of the corneal biometrics of the astigmatism group and the control group.

| Corneal biometrics (Mean ± SD) | Astigmatism group | Control group | *P* value |
| --- | --- | --- | --- |
| ISV | 21.10 ±8.68 | 16.75 ± 6.70 | < 0.001^*^ |
| IVA | 0.16 ± 0.08 | 0.15 ± 0.07 | < 0.001^*^ |
| KI | 1.01 ± 0.03 | 1.01 ± 0.03 | 0.931 |
| CKI | 1.00 ± 0.01 | 1.00 ± 0.01 | < 0.001^*^ |
| IHA | 6.43 ± 5.27 | 4.90 ±4.09 | < 0.001^*^ |
| IHD | 0.01 ± 0.01 | 0.01 ± 0.01 | < 0.001^*^ |
| Ecc | 0.53 ± 0.18 | 0.50 ± 0.17 | < 0.001^*^ |

SD = standard deviation, ISV = index of surface variance, IVA = index of vertical asymmetry, KI = keratoconus index, CKI = center keratoconus index, IHA = index of height asymmetry, IHD = index of height decentration, Ecc = anterior corneal eccentricity.

Independent two-sample *t* test, *P* value adjusted with the Bonferroni correction.

Supplementary Material Table 2. Correlations of the corneal biometrics with the axial length and the anterior corneal astigmatism.

| *r* (*P* value) | Axial length | Astigmatism |
| --- | --- | --- |
| ISV | -0.044 (< 0.001) | 0.393 (< 0.001) |
| IVA | -0.090 (< 0.001) | 0.081 (< 0.001) |
| KI | -0.044 (< 0.001) | - (0.171) |
| CKI | 0.107 (< 0.001) | 0.072 (< 0.001) |
| IHA | - (0.926) | 0.186 (< 0.001) |
| IHD | -0.046 (< 0.001) | 0.059 (< 0.001) |
| Ecc | -(0.550) | 0.105 (< 0.001) |

ISV = index of surface variance, IVA = index of vertical asymmetry, KI = keratoconus index, CKI = center keratoconus index, IHA = index of height asymmetry, IHD = index of height decentration, Ecc = anterior corneal eccentricity.

Pearson *r* correlation analyses.*r* values were presented only when *P* value< 0.05.

Supplementary Material Table 3. Distribution* of patients’ astigmatism types among the different astigmatism levels.

| Count(percentage in all) | | Type of astigmatism | | | Total |
| --- | --- | --- | --- | --- | --- |
|  |  | WTR | ATR | Oblique |  |
| Astigmatism  (D) | < 1 | 1558(23.09%) | 1727(25.60%) | 1046(15.50%) | 4331(64.19%) |
|  | 1-2 | 775(11.49%) | 1011(14.98%) | 197(2.92%) | 1983(29.39%) |
|  | 2-3 | 108(1.60%) | 220(3.26%) | 23(0.34%) | 351(5.20%) |
|  | ≥ 3 | 16(0.24%) | 62(0.92%) | 4(0.06%) | 82(1.22%) |
| Total | | 2457(36.42%) | 3020(44.76%) | 1270(18.82%) | 6747 |

**P*< 0.001 (Pearson’s χ^2^ test)

WTR = with-the-rule astigmatism, ATR = against-the-rule astigmatism, Oblique = oblique astigmatism.

Supplementary Material Table 4. Distribution* of patients’ astigmatism types among different axial lengths.

| Count(percentage in all) | | Type of astigmatism | | | Total |
| --- | --- | --- | --- | --- | --- |
|  |  | WTR | ATR | Oblique |  |
| Axial length  (mm) | < 20 | 0(0%) | 2(0.03%) | 5(0.07%) | 7(0.10%) |
|  | 20-22 | 109(1.62%) | 136(2.02%) | 68(1.01%) | 313(4.64%) |
|  | 22-24.5 | 1451(21.51%) | 1594(23.63%) | 698(10.35%) | 3743(55.48%) |
|  | 24.5-26 | 318(4.71%) | 404(5.99%) | 164(2.43%) | 886(13.13%) |
|  | 26-28 | 225(3.33%) | 377(5.59%) | 111(1.65%) | 713(10.57%) |
|  | 28-30 | 154(2.28%) | 221(3.28%) | 90(1.33%) | 465(6.89%) |
|  | ≥ 30 | 200(2.96%) | 286(4.24%) | 134(1.99%) | 620(9.19%) |
| Total | | 2457(36.42%) | 3020(44.76%) | 1270(18.82%) | 6747 |

**P* value < 0.001 (Pearson’s χ^2^ test)

WTR = with-the-rule astigmatism, ATR = against-the-rule astigmatism, Oblique = oblique astigmatism.

Supplementary Material Table 5. Distributions of astigmatism types in different age groups.

| Count (percentage in all) | | Type of astigmatism | | | Total |
| --- | --- | --- | --- | --- | --- |
|  |  | WTR | ATR | Oblique |  |
| Age  (years) | <40 | 44(0.65%) | 439(6.51%) | 64(0.95%) | 547(8.11%) |
|  | 40-45 | 117(1.73%) | 437(6.48%) | 84(1.24%) | 638(9.46%) |
|  | 50-60 | 353(5.23%) | 741(10.98%) | 269(3.99%) | 1363(20.20%) |
|  | 60-70 | 986(14.61%) | 1003(14.87%) | 501(7.43%) | 2490(36.91%) |
|  | ≥70 | 957(14.18%) | 400(5.93%) | 352(5.22%) | 1709(25.33%) |
| Total | | 2457(36.42%) | 3020(44.76%) | 1270(18.82%) | 6747 |

*P*< 0.001 with χ^2^ test.

WTR = with-the-rule astigmatism; ATR = against-the-rule astigmatism; Oblique = oblique astigmatism.

Descriptions:

Supplementary Material Table 1 presented the conic coefficients of cataract patients with comparisons between the two groups.

Supplementary Material Table 2 presented the correlations of the corneal biometrics with the axial length and the anterior corneal astigmatism.

Supplementary Material Table 3 and 4 indicated the compositions of the astigmatism types

with the different astigmatism levels and axial lengths.

Supplementary Material Table 5 showed the differences in the proportions of WTR, ATR and oblique astigmatism in different age levels, indicating a shift from ATR to WTR with age.
